# Social cognition assessment in Chinese adults with mild cognitive impairment: a scoping review

**DOI:** 10.3389/fpubh.2026.1850550

**Published:** 2026-08-24

**Authors:** Yan Liu, Chungui Zheng, Jianting Cao, Qinyu Guo, Jieting Hu, Yufei Lin, Ziping Pang, Xinran Song, Tiantian Zhai, Fangping Dang

**Affiliations:** 1 School of Nursing, Lanzhou University, Lanzhou, China; 2 Xingtai Medical College, Xingtai, Hebei, China; 3 Lanzhou University First Hospital, Lanzhou, China; 4 Lanzhou University Second Hospital, Lanzhou, China

**Keywords:** assessment tools, clinical implications, mild cognitive impairment, scoping review, social cognition

## Abstract

**Background:**

Social cognition is a core cognitive domain that is impaired in mild cognitive impairment (MCI), yet the availability and utility of assessment tools for this population in China remain unclear.

**Objective:**

This scoping review aimed to systematically map the current evidence on social cognition assessment tools for Chinese older adults with MCI, and to identify gaps in the literature concerning cultural adaptation and psychometric validation.

**Methods:**

We systematically searched CNKI, CBM, Wanfang, Weipu, PubMed, and Web of Science up to 23 June 2026. We included original studies that assessed social cognition tools in Chinese MCI patients with controls, extracted data on study design, tools, domains, and results, and conducted a narrative synthesis.

**Results:**

Six studies met the inclusion criteria, comprising 198 MCI participants and 197 healthy controls. The assessment tools covered three social cognition domains: empathy, facial emotion recognition, musical emotion recognition, and social perception. All studies reported significant impairments in MCI, particularly in negative emotion recognition (disgust, sadness, fear) and empathy. However, none reported reliability coefficients (e.g., Cronbach's α) or established diagnostic cut-off scores for the Chinese MCI population. Sample sizes were small (range 19–59 per MCI group), and all studies were single-center.

**Conclusion:**

Evidence suggests that Chinese MCI patients show social cognition deficits across multiple domains, yet current tools are insufficiently validated and culturally adapted. Future studies may consider multi-center validation, establishing normative data and cut-offs, and developing culturally relevant instruments for China.

## 1 Introduction

Mild cognitive impairment (MCI) is a condition characterized by progressive decline in memory or other cognitive functions that does not impair the ability to perform daily activities and does not meet diagnostic criteria for dementia (1). MCI can precede dementia by up to 20 years, and approximately 10%−15% of people with MCI progress to dementia annually (2). If MCI is assessed and identified at an early stage, and effective preventive measures and interventions are implemented, patients' cognitive function may be restored to a healthy level (3). Early screening for MCI relies on neuropsychological assessment, and social cognition—one of the six core cognitive domains—has gradually gained increasing attention from researchers (4, 5). Social cognition is a complex, multidimensional concept defined in psychology as the way individuals understand and think about others, forming inferences about people or objects based on social information in the environment; this process includes making inferences and judgements regarding others' mental states, behavioral motivations, and intentions (6). Social cognition first emerged in research on schizophrenia (7) and has since been applied to other conditions, including depression (8), bipolar disorder (9), autism (10, 11) and Alzheimer's disease (AD) (12).

Research has found that approximately 27.5% of patients with MCI exhibit varying degrees of social cognitive impairment, manifested specifically as deficits in emotion recognition, theory of mind (ToM), and empathy (13). Social cognitive impairment may precede memory difficulties; people with non-amnestic MCI may show deficits in ToM as early as 6–12 months before the onset of executive dysfunction (14). A systematic review by Roheger et al. indicates that ToM and emotional cognition can predict the progression from MCI to AD; individuals with ToM deficits face a 1.5- to 2-fold increased risk of developing AD within 3 years (15). It is therefore clear that accurate assessment of social cognition is of great significance for the screening, prevention, and early intervention of MCI. Accordingly, this scoping review systematically maps the current landscape of social cognition assessment tools used in Chinese MCI populations, with the goal of informing clinical practitioners and researchers about the existing evidence base, identifying critical gaps in cultural adaptation and psychometric validation, and providing direction for the future development of culturally appropriate assessment instruments for MCI in China.

## 2 Methods

### 2.1 Literature search

We searched PubMed, Web of Science, CNKI, CBMdisc, China Science and Technology Journal Database, and Wanfang Data for citations published from inception to 23 June 2026, without language restrictions. We additionally reviewed the reference lists of included studies and relevant systematic reviews. Two reviewers (LYF and SXR) independently conducted the literature search, with disagreements resolved by consensus or by consulting a third reviewer (PZP) (16). Search terms were developed by the research team to encompass three main domains: assessment tool, social cognition, and mild cognitive impairment.

### 2.2 Eligibility criteria

Studies were included if they: (1) were original studies that primarily aimed to examine the applicability, psychometric properties, or clinical utility of a social cognition assessment tool in older adults with MCI (including amnestic MCI, Parkinson's disease with MCI, and vascular cognitive impairment no dementia); (2) included Chinese participants; (3) included a healthy control group; (4) reported at least one comparison of social cognition performance between the MCI and control groups; and (5) were peer-reviewed articles published in English or Chinese.

Studies were excluded if they: (1) were reviews, conference abstracts, case reports, or other non-original research; (2) used assessment tools that did not primarily measure social cognition (e.g., tools assessing memory, executive function, attention, visuospatial ability, general cognition, or eye-tracking parameters); or 3) did not report sufficient data to extract MCI-control comparisons on the social cognition measure.

### 2.3 Data extraction

A detailed data-extraction form was developed to capture key information from each included study, including first author and year, study location, assessment tool, social cognition domain assessed, sample size (MCI/HC), and mean age of the MCI group. Information pertaining to each tool's reported psychometric properties, administration time, strengths, and limitations was also collected. In addition, we extracted key findings regarding group comparisons between MCI and healthy controls on social cognition measures, as well as any psychometric data reported (e.g., reliability, validity, area under the curve [AUC], sensitivity, specificity), where available.

### 2.4 Data analysis

We therefore adopted a narrative synthesis approach, following the analytical steps below. First, we compiled all extracted data into a summary table, capturing the following key study characteristics: references, study location, assessment tool, social cognition domain, sample size (MCI/HC), mean age of MCI group, and principal findings. Second, we classified studies according to their targeted social cognition subdomain. Drawing on the conceptual framework of social cognition, we grouped studies into three thematic categories: empathy, emotion recognition, and social perception. Within each category, we synthesized findings to identify common patterns and discrepancies. Third, for each study, we extracted key results comparing MCI and healthy control groups. Psychometric properties were recorded when available and explicitly noted as “not reported” when absent. Finally, to contextualize the evidence within China, we compared our findings with those from large-scale population-based validation studies conducted in non-Chinese samples. This comparison served as a methodological reference, highlighting existing gaps in the Chinese literature.

## 3 Results

### 3.1 Study selection

**Figure 1:**
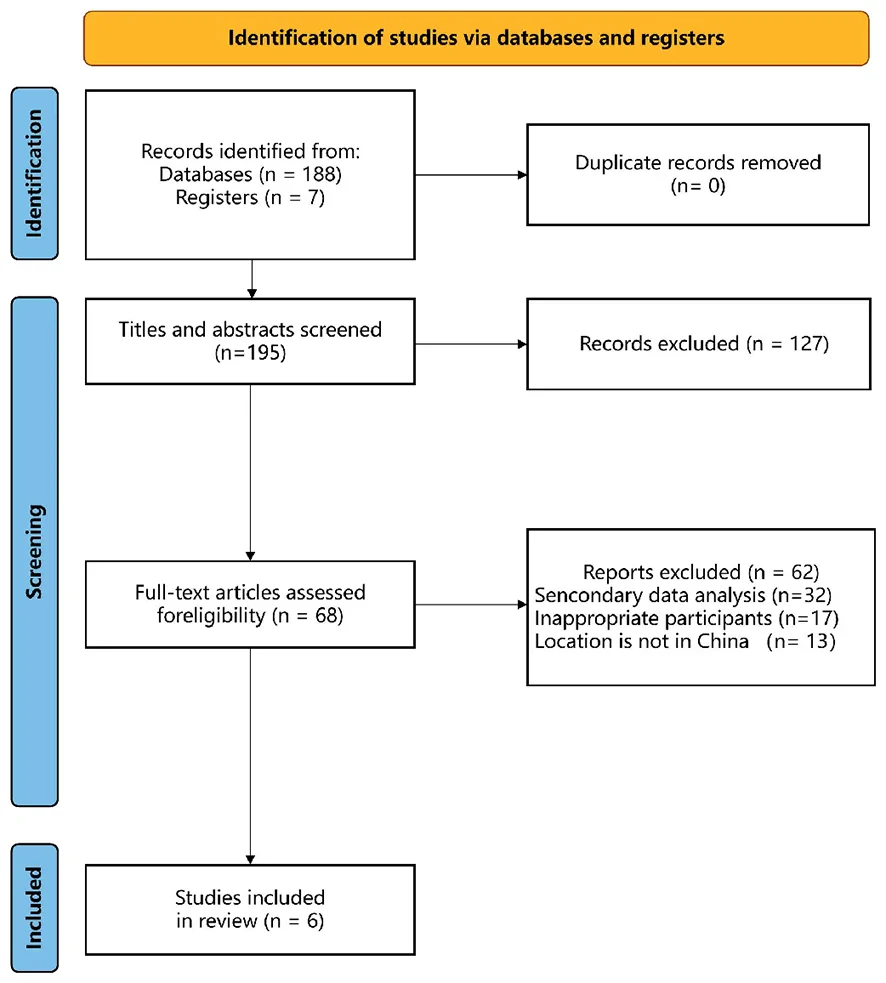
Study selection.

The initial database search yielded 195 records, which were screened by title and abstract. Of these, 68 full-text articles were assessed for eligibility. A total of six studies met the inclusion criteria and were included in this review. The study selection process is shown in the PRISMA flow diagram (Figure 1).

### 3.2 Study characteristics

A summary of the six included studies is presented in Table 1. All studies were cross-sectional and employed a case-control design comparing a clinically diagnosed MCI group with a cognitively healthy control group. Sample sizes ranged from 19 to 59 for MCI groups (total 198 participants across studies) and 16 to 63 for control groups (total 197 participants). Studies were conducted in Hefei, Bozhou, Tianjin, and Taipei. Mean MCI age ranged from 58.6 to 70.0 years. MCI subtypes included aMCI, PD-MCI, VCIND, and unspecified MCI. Assessment tools covered three social cognition subdomains: empathy, emotion recognition, and social perception.

**Table 1:** Characteristics of included studies.

References	Location	Assessment tool	Social cognition domain	Sample size (MCI/HC)	Mean age (MCI)	Key findings	Psychometric properties reported	Administration time	Strengths	Limitations
Zhang (17)	Anhui	IRI-C	Empathy	25/25	69.0 ± 8.2	MCI group showed significantly lower IRI total score, Empathic Concern (EC), and Personal Distress (PD) subscale scores compared to HC	NR	NR	First study to demonstrate empathy deficits in Chinese MCI using a validated Western scale; simple and quick to administer	Small sample size; single-center; no psychometric validation in MCI sample; no cut-off scores established
Wu et al. (18)	Anhui	IRI-C + BES	Empathy	22/21	65.6 ± 6.2	MCI group showed significantly lower IRI total, Perspective Taking (PT), and Fantasy (FS) scores; lower BES total (particularly emotional empathy); higher AES-I apathy scores; PD and apathy scores correlated negatively with gray matter volume	NR	NR	Combined assessment of empathy (IRI-C) and emotional empathy (BES); included neuroimaging correlates (VBM); assessed apathy as a related construct	Small sample size; single-center; no psychometric validation in MCI sample; no cut-off scores established; VBM analysis underpowered
Chiang et al. (19)	Taiwan	MMER app (facial emotion recognition)	Facial emotion recognition	42 (PD-MCI)/63	~70	PD-MCI group showed selective impairment in recognizing “disgust”; attention partially mediated the relationship between executive function and disgust recognition	NR	NR	Computerized, tablet-based assessment; used East Asian facial stimuli addressing the “own-race effect”; identified specific emotion (disgust) and mediating cognitive mechanism	PD-MCI sample not generalizable to all MCI subtypes; no psychometric validation in MCI sample; no cut-off scores established
Zhou et al. (20)	Anhui	Musical emotion recognition test	Musical emotion recognition	19 (aMCI)/16	69.5 ± 8.9	aMCI group showed selective impairment in recognizing “fear” in music; fear recognition correlated positively with MMSE scores and memory performance (AVLT)	NR	NR	First study to assess emotion recognition in the auditory (music) domain in Chinese aMCI; cross-modal evidence complements facial emotion recognition studies	Very small sample size; single-center; no psychometric validation in MCI sample; no cut-off scores established; stimuli were Western classical music excerpts
Wang et al. (21)	Tianjin	FIM (social cognition subdomain)	Social perception	59 (VCIND)/45	58.6 ± 6.3	VCIND group showed significantly lower scores on FIM social cognition and communication subdomains; FIM total correlated positively with MMSE and MoCA	NR	NR	Largest MCI sample among included studies (*n* = 59); FIM is a widely used clinical tool; social cognition subdomains can be easily integrated into routine clinical assessment	VCIND sample not generalizable to all MCI subtypes; mean age (58.6) younger than typical MCI population; social cognition subdomains are not a standalone validated social cognition measure; no psychometric validation in MCI sample
Chuang et al. (22)	Taiwan	Taiwan facial emotion recognition task	Facial emotion recognition	31 (aMCI)/27	70.0 ± 6.9	aMCI and AD groups showed impaired recognition of “sadness”; mislabeled younger faces as “anger” and older faces as “neutral”; ROC analysis: AUC = 0.77–0.80 for SCD vs. MCI; sensitivity = 81–91%, specificity = 39%	**Reliability:** Not reported; **Validity:** ROC analysis reported (SCD vs. aMCI: AUC = 0.77–0.80, Sen = 81–91%, Spe = 39%)	NR	Used East Asian facial stimuli (Taiwanese faces) addressing the “own-race effect”; ROC analysis provides preliminary diagnostic utility data; included SCD, aMCI, and AD groups allowing comparison across the cognitive continuum	HC group significantly younger than MCI group (62.5 vs. 70.0 years), potentially inflating group differences; low specificity (39%) limits clinical utility; small sample size; single-center; authors acknowledged “lack of reliability and validity”

IRI-C, Interpersonal Reactivity Index-Chinese version; BES, Basic Empathy Scale; FIM, Functional Independence Measure; MMSE, Mini-Mental State Examination; MoCA, Montreal Cognitive Assessment; AES-I, Apathy Evaluation Scale-Informant version; VBM, voxel-based morphometry; PD-MCI, Parkinson's disease with mild cognitive impairment; aMCI, amnestic mild cognitive impairment; VCIND, vascular cognitive impairment no dementia; HC, healthy control; AD, Alzheimer's disease; SCD, subjective cognitive decline; ROC, receiver operating characteristic; AUC, area under the curve; Sen, sensitivity; Spe, specificity; AVLT, auditory verbal learning test; NR: Not reported.

### 3.3 Social cognition findings by assessment tool

#### 3.3.1 Empathy

Two studies examined empathy in MCI using the IRI-C, with one also employing the BES. Zhang et al. compared 25 MCI patients with 25 healthy controls and found significantly lower IRI total scores in the MCI group (40.30 ± 7.20 vs. 51.27 ± 4.43, *p* < 0.05), as well as lower scores on the Empathic Concern (EC) and Personal Distress (PD) subscales (EC: 14.30 ± 1.60 vs. 15.13 ± 2.64; PD: 1.98 ± 2.13 vs. 7.71 ± 3.40). These findings suggest that MCI patients exhibit broad empathy deficits affecting both cognitive and affective components.

Wu et al. extended these findings in a sample of 22 MCI patients, reporting similar results on the IRI-C and additionally showing significantly lower BES total scores, particularly on the emotional empathy subscale. Moreover, the MCI group had significantly higher apathy scores on the AES-I. Correlation analyses revealed that IRI-PD and apathy scores were negatively correlated with gray matter volume, while emotional empathy scores were positively correlated with gray matter volume, suggesting a neural basis for empathy impairment in MCI.

#### 3.3.2 Facial emotion recognition

Two studies assessed facial emotion recognition in MCI using Taiwanese facial expression stimuli. Chiang et al. administered a computerized facial emotion recognition task (MMER app) to 42 PD-MCI patients and 63 healthy controls. Results showed that PD-MCI patients exhibited a selective impairment in recognizing disgust expressions, while recognition of other basic emotions was relatively preserved. Mediation analysis indicated that attention partially mediated the relationship between executive function and disgust recognition, suggesting that cognitive processes contribute to emotion recognition deficits in MCI.

Chuang et al. administered the Taiwan Facial Emotion Recognition Task to 31 aMCI patients, 18 very mild AD patients, and 27 healthy older adults. Both patient groups showed significantly impaired recognition of sadness compared with healthy controls, whereas recognition of other emotions (happiness, anger, surprise, fear) remained relatively intact. Notably, aMCI and AD patients tended to mislabel sadness as anger when viewing younger faces and as neutral when viewing older faces, suggesting a complex interaction between emotion recognition and face age. Although the own-age effect was not statistically significant, a trend toward better recognition of older faces was observed in patient groups. ROC analysis for discriminating SCD from aMCI yielded AUCs of 0.80 for younger faces and 0.77 for older faces (sensitivity: 81–91%; specificity: 39%), indicating that sadness recognition may have potential as a screening indicator but with limited specificity.

#### 3.3.3 Musical emotion recognition

One study investigated emotion recognition in the auditory domain. Zhou et al. administered a musical emotion recognition test to 19 aMCI patients, 16 mild-to-moderate AD patients, and 16 healthy controls. Participants were asked to identify one of four emotional labels (happy, peaceful, sad, fearful) from 40 instrumental musical excerpts. Results showed that both patient groups exhibited a selective deficit in recognizing fear in music compared with healthy controls, while recognition of happy, peaceful, and sad emotions was relatively preserved. Correlation analyses demonstrated that fear recognition accuracy was positively correlated with MMSE scores (r = 0.578, *p* < 0.001) and with memory task performance (AVLT immediate recall: r = 0.572; delayed recall: r = 0.623), suggesting that fear recognition in music shares common cognitive substrates with general cognitive function and memory.

#### 3.3.4 Social perception

One study examined social perception using the social cognition subdomains of a functional independence measure. Wang et al. administered the Functional Independence Measure (FIM) to 59 VCIND patients and 45 healthy controls. The FIM includes social cognition and communication subdomains in addition to motor function items. Results showed that VCIND patients scored significantly lower than healthy controls on the social cognition subdomain (11.68 ± 2.68 vs. 4.36 ± 1.05) and the communication subdomain (13.25 ± 1.75 vs. 5.86 ± 1.12), while motor function subdomains (self-care, sphincter control, transfers, locomotion) did not differ significantly between groups. FIM total scores correlated positively with MMSE (r = 4.556, *p* < 0.001) and MoCA scores (r = 3.047, *p* = 0.004), suggesting that social cognitive decline in VCIND is associated with general cognitive impairment severity.

## 4 Discussion

### 4.1 Main characteristics of social cognitive impairments in Chinese MCI patients

The six studies consistently demonstrated that Chinese MCI patients exhibit impairments across multiple social cognitive domains, including empathy, emotion recognition, and social perception. Regarding empathy, both cognitive (perspective-taking) and affective (empathic concern and personal distress) components were impaired, suggesting that empathy deficits in this population are broad rather than domain-specific. For emotion recognition, deficits in negative emotions—particularly disgust, sadness, and fear—were the most prominent findings across both visual (facial) and auditory (musical) modalities. This pattern of selective negative emotion recognition impairment is broadly consistent with international findings in MCI and early AD (23, 24) suggesting that negative emotion processing may be particularly vulnerable to early neurodegenerative changes. Notably, the presence of social cognitive impairments at the MCI stage, before clinically significant functional decline, suggests that social cognition measures may hold potential as early screening indicators for individuals at risk of dementia progression.

### 4.2 Cultural adaptation gaps in Chinese assessment tools

The most prominent issue identified in this review is the severe lack of culturally adapted validation for social cognition assessment tools in Chinese MCI populations. Among the six included studies, the IRI-C, BES, and FIM are direct Chinese translations or adaptations of Western instruments, with item content, scoring criteria, and social scenarios rooted in Western cultural contexts. For example, the empathic concern items of the IRI-C describe situations embedded in Western individualistic frameworks of emotional expression, which may be interpreted differently in the Chinese collectivist context, where emotional restraint and relational harmony are often prioritized. Similarly, although some facial emotion recognition studies (19, 22) have adopted East Asian facial stimuli to mitigate the “own-race effect,” their emotion classification systems remain grounded in Ekman's six basic emotion model—a framework whose cross-cultural universality has been increasingly questioned (25, 26).

A more critical concern is the pervasive absence of psychometric validation across all included studies. None of the six studies reported reliability coefficients (e.g., Cronbach's α or ICC) for the assessment tools in their MCI samples, nor did they establish diagnostic cut-off scores or screening validity metrics (AUC, sensitivity, specificity) specifically for Chinese MCI populations.

Furthermore, all included studies were single-center investigations with small sample sizes (MCI groups ranging from 19 to 59 participants), concentrated in a limited number of regions (Anhui, Tianjin, and Taiwan). The absence of multi-center, large-sample data severely limits the generalisability of the findings. Notably, Wang et al. focused on a VCIND population with a mean age of 58.6 years, which deviates from the typical MCI population (>65 years), potentially affecting the external validity of its results (21).

### 4.3 Comparative methodological lessons

In stark contrast to the Chinese evidence base, several countries have conducted large-scale or well-controlled validation studies of social cognition assessment tools in older adult populations. Lee et al., using the US-based MYHAT population cohort (*n* = 902), established a social cognition composite score comprising the SNQ-22 and the RMET-10, and systematically evaluated its reliability, associations with other cognitive domains, AUC for discriminating MCI (CDR = 0.5; AUC = 0.615), and its impact on MCI classification rates (27). In the Netherlands, Kessels et al. compared facial emotion recognition across aMCI, AD, and healthy controls, finding that aMCI patients showed significantly lower accuracy than controls, with impairment correlating with greater functional dependence (28) —demonstrating that social cognition deficits in MCI have tangible real-world consequences. Furthermore, a meta-analysis by Velakoulis D et al. (23) synthesized evidence across multiple neurodegenerative disorders and confirmed that emotion recognition and theory of mind impairments are consistently present in early AD and MCI, with negative emotions (particularly fear and sadness) being most affected—a pattern that closely mirrors the findings from the Chinese studies in our review. Although the specific instruments (RMET-10, Ekman 60 Faces, Faux Pas Test) are Western-derived, their research paradigms—large samples, multi-center designs, systematic psychometric reporting, and cross-diagnostic comparisons—provide methodological benchmarks for Chinese research.

### 4.4 Strengths and limitations

This review has several strengths. It is, to our knowledge, the first scoping review to systematically synthesize evidence on social cognition tools for Chinese MCI populations, providing a foundational overview of this emerging field. A systematic search of six English and Chinese databases ensured broad literature coverage. Furthermore, the narrative synthesis not only summarizes existing evidence but also highlights key methodological and cultural gaps for future research and tool development.

However, several limitations of this review should also be acknowledged. First, the review was restricted to English and Chinese language publications, which may introduce language bias and potentially omit relevant studies published in other languages. Second, due to the limited number of included studies and their substantial heterogeneity in terms of assessment tools, MCI subtypes, and outcome measures, a meta-analysis was not feasible. Third, age matching between MCI and control groups was suboptimal in some included studies; for example, Chuang et al. (22) had a significantly younger healthy control group compared to the MCI group, which may have inflated group differences in emotion recognition performance. Fourth, as a scoping review, formal quality assessment of the included studies was not performed. Finally, publication bias may have influenced the available evidence, as studies reporting null findings may be less likely to be published, potentially overrepresenting positive findings in the literature.

### 4.5 Future research directions

Based on these findings, future research may consider three directions. First, develop culturally grounded tools: Chinese-normed facial emotion materials, empathy and faux pas tasks reflecting Chinese social norms, and theory of mind measures with culturally relevant scenarios. Second, conduct large-scale validation: multi-center studies to establish norms, cut-offs, and screening metrics (sensitivity, specificity, and AUC) for Chinese MCI populations. Third, integrate multiple subdomains—empathy, emotion recognition, and ToM—into a comprehensive social cognition assessment system for MCI (29, 30).

## 5 Conclusions

This scoping review systematically examined social cognition assessment tools for MCI in China. The six included studies consistently show that Chinese MCI patients have impairments across empathy, emotion recognition (facial and musical), and social perception. Yet all studies share limitations: small samples, insufficient cultural adaptation, and no psychometric validation (reliability, cut-offs, or screening metrics). Compared with international population-based validation studies, China shows a substantial methodological gap. The lack of validated tools hampers early identification and clinical intervention for social cognitive impairments in MCI. Future research could focus on culturally adapted tools grounded in Chinese social contexts, alongside multi-center, large-sample validation to establish national norms and diagnostic standards.
